# Potential Therapeutic Value of a Novel FAAH Inhibitor for the Treatment of Anxiety

**DOI:** 10.1371/journal.pone.0137034

**Published:** 2015-09-11

**Authors:** Eva M. Marco, Cinzia Rapino, Antonio Caprioli, Franco Borsini, Giovanni Laviola, Mauro Maccarrone

**Affiliations:** 1 Section of Behavioural Neuroscience, Dept. Cell Biology and Neurosciences, Istituto Superiore di Sanità, Rome, Italy; 2 Dept. Biomedical Sciences, Università degli Studi di Teramo, Teramo, Italy; 3 Sigma-Tau R&D, Pomezia, Italy; 4 School of Medicine, Campus Bio-Medico University of Rome, Rome, Italy; 5 Centro Europeo di Ricerca sul Cervello (CERC)/IRCCS Fondazione Santa Lucia, Rome, Italy; University of Parma, ITALY

## Abstract

Anxiety disorders are among the most prevalent psychiatric diseases with high personal costs and a remarkable socio-economic burden. However, current treatment of anxiety is far from satisfactory. Novel pharmacological targets have emerged in the recent years, and attention has focused on the endocannabinoid (eCB) system, given the increasing evidence that supports its central role in emotion, coping with stress and anxiety. In the management of anxiety disorders, drug development strategies have left apart the direct activation of type-1 cannabinoid receptors to indirectly enhance eCB signalling through the inhibition of eCB deactivation, that is, the inhibition of the fatty acid amide hydrolase (FAAH) enzyme. In the present study, we provide evidence for the anxiolytic-like properties of a novel, potent and selective reversible inhibitor of FAAH, ST4070, orally administered to rodents. ST4070 (3 to 30 mg/kg *per os*) administered to CD1 male mice induced an increase of time spent in the exploration of the open arms of the elevated-plus maze. A partial reduction of anxiety-related behaviour by ST4070 was also obtained in *Wistar* male rats, which moderately intensified the time spent in the illuminated compartment of the light-dark box. ST4070 clearly inhibited FAAH activity and augmented the levels of two of its substrates, *N*-arachidonoylethanolamine (anandamide) and *N*-palmitoylethanolamine, in anxiety-relevant brain regions. Altogether, ST4070 offers a promising anxiolytic-like profile in preclinical studies, although further studies are warranted to clearly demonstrate its efficacy in the clinic management of anxiety disorders.

## Introduction

Anxiety disorders are among the most prevalent psychiatric diseases with high personal costs and a remarkable socio-economic burden. However, current treatment is far from satisfactory [see [1,2] for recent updates on this topic]. In recent years, the endocannabinoid (eCB) system has received special attention as a potential pharmacological target for the management of anxiety disorders, given the increasing body of evidence (from both human and animal studies) that supports its central role in emotional control [3,4,5]. The eCB system mainly consists in metabotropic membrane receptors, their endogenous ligands (eCBs), and the enzymes responsible for their synthesis and degradation. Endocannabinoids, mainly N-arachidonoylethanolamine (anandamide, AEA) and 2-arachidonoylglycerol (2-AG), are synthesized ‘on demand’ by the cleavage of membrane phospholipid precursors. The eCB signalling ends by the enzymatic activity of specific enzymes, fatty acid amide hydrolase (FAAH) in charge of AEA hydrolysis and monoacylglycerol lipase (MAGL) responsible for the hydrolysis and inactivation of 2-AG [see [6,7] for more detailed information]. The extensive research on the eCB system has provided multiple targets for the pharmacological manipulation of this neuromodulatory system. Despite the direct activation or blockade of the specific metabotropic receptors was the initial approach, the indirect enhancement of the eCB signalling has become more popular given its spatiotemporal specificity as well as the reduced adverse effects observed [8,9]. In recent years, several FAAH inhibitors have been developed, i.e. URB597, AACOCF3 or PF-3845 [10,11], and have been associated with anxiolytic, antidepressant and analgesic properties in rodents [12,13,14]. More recently, a new family of oxime carbamates has been identified as potent inhibitors of FAAH [15,16], and here we sought to interrogate the putative anxiolytic-like profile of the enol-carbamate 1-biphenyl-4-ylethenyl piperidine-1-carboxilate, ST4070. To this aim, rodents orally administered ST4070 were evaluated in behavioural paradigms widely used for preclinical screening of anxiolytic drugs. FAAH activity and eCB content in specific brain regions related to emotionality, motivation and behavioural planning (hippocampus, striatum and frontal cortex) [17] were also assessed, in order to better understand the neurobiological mechanisms through which ST4070 acts to modulate anxiety.

## Material and Methods

### 1. Animals

This study was carried out in strict accordance with the guidelines of the European Communities (2010/63/UE) regulating animal research. The protocol was approved by the Italian Ministry of Health’s dedicated Committee (N° SSA/252/14).

Male CD1 mice and *Wistar* rats were purchased from Charles River (Milan, Italy). Mice (n = 60), around 35 g at arrival, were housed four per cage in Macrolon II cages (26.7 cm x 20.7 cm x 14 cm height), with stainless steel feed racks and sterilized, dust-free bedding cobs in the *Sigma-Tau* animal facilities. The room was maintained at constant temperature (22 ± 2°C) and relative humidity (55 ± 10%). A circadian 12-hour cycle of artificial light was maintained (lights on at 7 a.m.). Rats (n = 50), around 250 g at arrival, were paired-housed in Macrolon III plexiglas cages (42 x 26.5 x 18.5 cm height) at the *Istituto Superiore di Sanità* animal facilities. The room was maintained at constant temperature (22 ± 2°C) and relative humidity (60 ± 10%). A circadian 12-hour cycle of artificial light was maintained (lights on at 8 a.m.). All cages were placed in racks that allowed the animals to see, hear, and smell other animals. All animals had food pellet diet (Mucedola, Italy) and tap water *ad libitum*.

### 2. Drugs and chemicals

The fatty acid amide hydrolase (FAAH) inhibitor 1-biphenyl-4-ylethenyl piperidine-1-carboxylate (ST4070) was kindly provided by Sigma-Tau (Italy) [15,16]. ST4070 was dispersed daily in a solution containing 0.5% Carboxymethylcellulose sodium salt (CMC, medium viscosity- Sigma, Milan, Italy) and 0.1% Tween 80 (Merck, Darmstadt, Germany) in distilled water. Diazepam (FIS, Vicenza, Italy) was dispersed daily in a solution containing 3% Tween-80 in sterile water, and was used as a reference compound.

### 3. Evaluation of anxiety-related behaviours

Anxiety-related behaviours were measured in two different anxiety tests based on unconditioned responses—the elevated plus maze, EPM [18] and the light-dark box, LD box [19]; evaluation in these tests requires no training and usually has a high eco/ethological validity (see [20,21] for review). Moreover, both tests are extensively employed nowadays for the preclinical screening of anxiolytic drugs in rodents. In this study we specifically used the EPM for the evaluation of mice [22] and the LD box for rats [23].

#### 3.1. Elevated-plus maze in mice

The apparatus made of grey Plexiglas consisted of two open and two closed arms linked by a common central platform. The maze was elevated 40 cm above floor level and dimly lighted. Animals were individually placed on the central platform of the maze facing an open arm. A standard 5-min test was employed [18]. The amount of time spent by each animal in either open or closed arm was recorded by an any-maze video tracking system (Ugo Basile, Milan, Italy), and so was the number of entries and the total distance walked by each animal into either arm.

#### 3.2. Dark-light test in rats

The apparatus made in opaque Plexiglas consisted of two compartments, of which one was brightly lighted, and was placed in a sound-attenuating chamber. The box compartments (45 cm x 30 cm x 35 cm) were distinguished only by wall colour and illumination. The dark compartment had black walls, whereas the lit compartment had white walls. A guillotine door separated the two compartments. The lit compartment (450 lux) was illuminated by a desk lamp placed over the compartment itself. The location of each rat was monitored with photocells and scored by software using a computer that was interfaced with the boxes. The photocells were located a few cm from the floor along the walls of the box. Crossing from one compartment to the other was scored whenever the rat emerged far enough from one side to interrupt the first photocell beam, while no longer interrupting the photocell beams in the original compartment. Time spent in the lit compartment and entries made to the lit compartment were recorded and considered as the most significant parameters for the evaluation of anxiety, while beam interruptions in the dark compartment were used as a measure of activity rate [19].

### 4. Experimental design

#### Experiment 1: Effects of ST4070 administration in mice exposed to the elevated plus maze

Animals remained for at least seven days before the beginning of the experiment (period of acclimatization) in the animal facilities. Sixty min before the test mice were orally administered ST4070 at doses of 3, 10 and 30 mg/10 mL/kg (12 mice/group). Diazepam was intraperitoneally administered 30 min before the test at a dose of 1 mg/5 ml/kg (12 mice/group). Range of drug dosage and schedule of administration were based on previous experiments [15]. Animals were individually placed in the elevated plus-maze to begin the 5-min test session. Behavioural testing was performed between 09.00 and 14.00 and animals were randomly assigned to the different drug groups.

#### Experiment 2: Effects of ST4070 administration in rats exposed to the light-dark test

Animals remained for at least seven days before the beginning of the experiment (period of acclimatization) in the animals’ facilities. Sixty min before the test rats were orally administered ST4070 at doses of 10 and 30 mg/2 mL/kg (16–18 rats/group). Range of drug dosage and schedule of administration were based on previous pilot experiments performed in our laboratory. Animals were individually placed in the illuminated compartment to begin the 10-min test session in the LD box. Behavioural testing was performed between 09.00 and 14.00 and animals were randomly assigned to the different drug groups. Immediately after testing, animals were sacrificed by decapitation, brains were rapidly removed and discrete brain regions were dissected on ice and stored at -80°C until biochemical assays.

### 5. Endocannabinoid system analysis

Frontal cortex, striatum and hippocampus from 8 animals per experimental group were randomly selected to evaluate particular components of the endocannabinoid system. Since we have previously demonstrated that ST4070 shows remarkable selectivity for FAAH over other components of the endocannabinoid system [16], we focused our investigation on the evaluation of FAAH activity, as well as in the measurement of endogenous cannabinoid ligands targeted by this enzyme, namely anandamide (N-arachidonoylethanolamine, AEA) and palmitoylethanolamide (PEA) and 2-arachidonoylglycerol (2-AG).

The hydrolysis of 10 μM AEA-ethanolamine-1-[^3^H] (60 Ci/mmol) by FAAH was assayed in brain extracts (50 μg protein/test), by measuring the release of [^3^H]ethanolamine as reported [15]. FAAH activity was expressed as pmol product formed per min per mg protein (pmol/min per mg protein).

For the measurement of endocannabinoid levels, brain tissues were subjected to lipid extraction with chloroform/methanol (2:1, v/v), in the presence of d_8_-AEA, d_8_-2-AG and d_4_-PEA as internal standards. The organic phase was dried and then analysed by liquid chromatography-electrospray ionization mass spectrometry (LC–ESI-MS), using a single quadrupole API-150EX mass spectrometer (Applied Biosystem, CA, USA) in conjunction with a PerkinElmer LC system (PerkinElmer, MA, USA). Quantitative analysis was performed by selected ion recording over the respective sodiated molecular ions, as reported [24]. Chemicals were of the purest analytical grade. AEA and PEA were purchased from Sigma Chemical Co. (St. Louis, MO, USA). 2-AGwas from ALEXIS (San Diego, CA, USA). AEA-ethanolamine-1-[^3^H] (60 Ci/mmol) was purchased from PerkinElmer Life Sciences (Boston, MA). d8-AEA, d8-2-AG and d4-PEA were from Cayman Chemicals (Ann Arbor, MI, USA).

### 6. Data analysis

Data, expressed as mean ± SEM, were controlled for normality (Kolmogorov-Smirnoff test) and homogeneity of variances (Levene test). In case normality failed, data were transformed accordingly. Differences between groups were evaluated using a one-way analysis of variance (ANOVA). Tukey honestly statistical difference was employed as the *post-hoc* comparison test and statistical significance was considered at a p-level value ≤ 0.05. Statistical analyses were performed by using the IBM SPSS Statistics 19 software (IBM Corporation, New York, USA).

## Results

### Effects of ST4070 on anxiety-related responses

**Fig 1:**
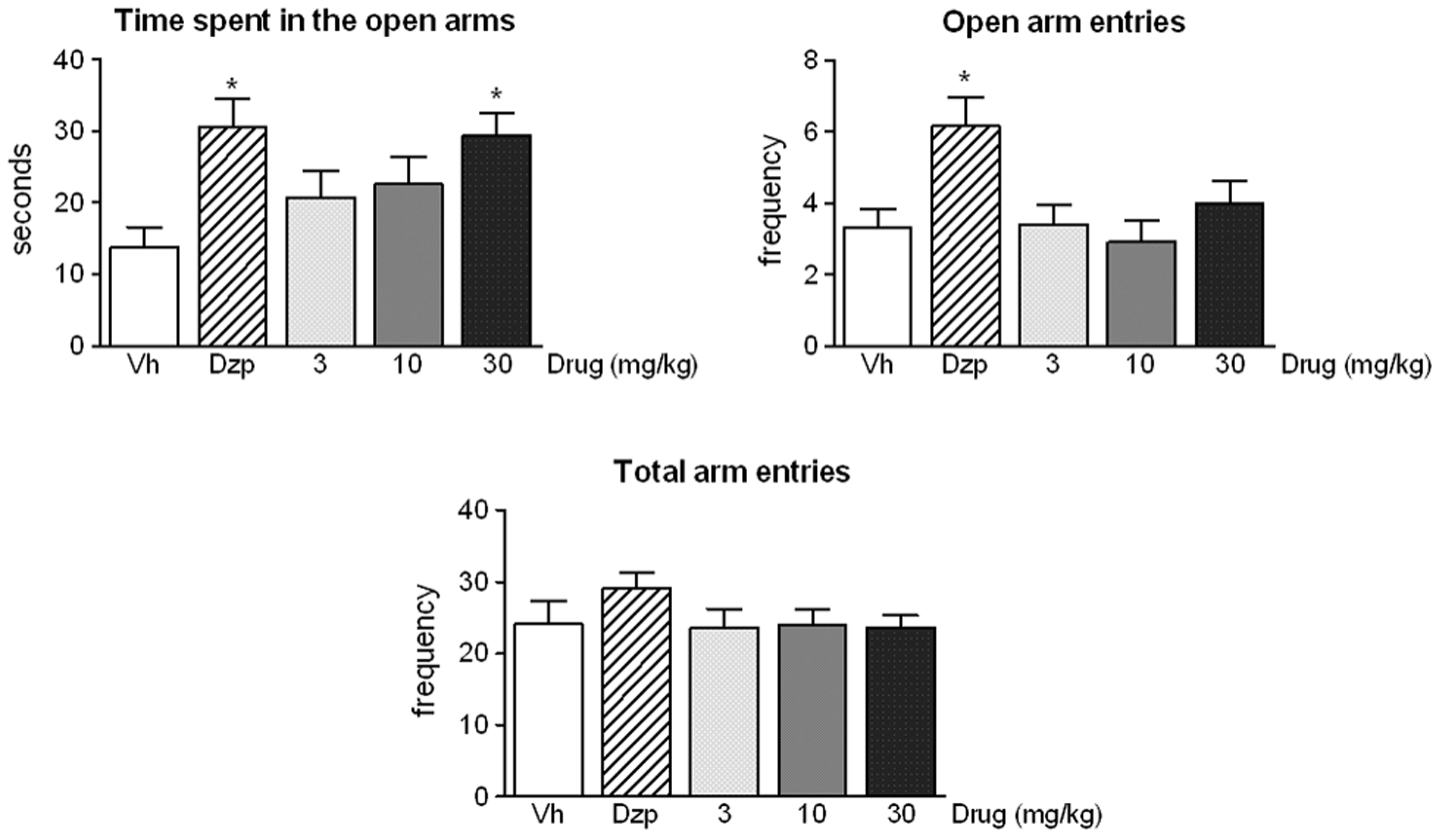
ST4070 effects in mice exposed to the elevated plus maze. Data are expressed as mean ± SEM. Animals were orally administered ST4070 (3, 10 or 30 mg/kg, 60 min before testing) or diazepam (Dzp, 1 mg/kg, i.p. 30 min before testing), and were challenged in the elevated plus-maze for 5 min (n = 12 per experimental group). One-way ANOVA followed by Tukey *post-hoc* comparisons, * p < 0.05 *vs*. vehicle.

In the elevated plus-maze (Data in S1 Table), a significant drug effect was found for the time spent in the open arms [F(4,55) = 3.67, p<0.05] and for the number of entries into the open arms [F(4,55) = 4.18, p<0.01] (Fig 1, panels A and B). *Post-ho*c comparisons confirmed the expected reduction of anxiety-like behaviour induced in mice by diazepam, measured as the time spent in open arms and the frequency of entries into open arms. Similarly, the highest dose of ST4070 (30 mg/kg) also increased the time spent by animals in exploring the open arms, again suggesting a reduction in anxiety-like responses. No effects were found neither for diazepam nor for ST4070 on general locomotion, measured as the frequency of total arm entries [F(4,55) = 0.97, ns] (Fig 1, panel C).

**Fig 2:**
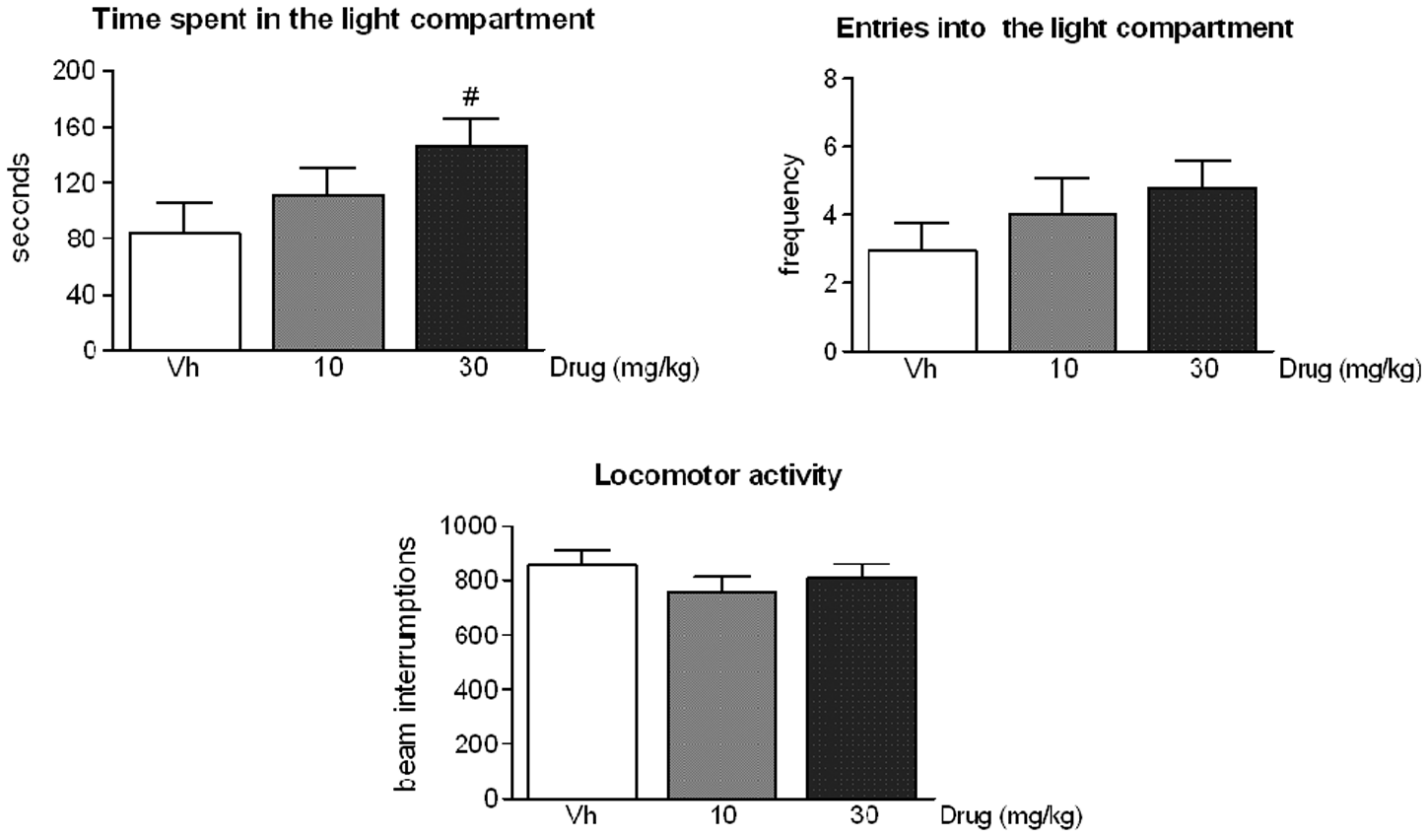
ST4070 effects in rats exposed to the light-dark box. Data are expressed as mean ± SEM. Rats were orally administered ST4070 (10 or 30 mg/kg, 60 min before testing), and were challenged in the LD box for 10 min (n = 16–18 per experimental group). One-way ANOVA followed by Tukey *post-hoc* comparisons, # p = 0.07 *vs*. vehicle.

In parallel, the highest dose of ST4070 (30 mg/kg) also seemed to increase the time spent by rats in the illuminated compartment of the LD box (Fig 2, panels A; Data in S2 Table). *Post-hoc* comparisons achieved an almost significant effect (p = 0.07) of the drug in the time spent by animals in exploring the light compartment, when compared to vehicle administered animals. No changes were observed in the number of transitions into the light compartment [F(2,47) = 1.08, ns] nor in general locomotor activity [F(2,47) = 0.78, ns] (Fig 2, panels B and C). It is noteworthy that the time spent in the light compartment seems to be a more sensitive parameter to the anxiolytic action of drugs than the number of transitions between lit and dark compartments of the apparatus [25,26].

### Effects of ST4070 on the eCB system

Present data further support the inhibitory action of ST4070 on FAAH activity (Data in S3 Table), since the oral administration of this compound to rats remarkably reduced FAAH activity in the specific brain areas analysed [frontal cortex: F(2,21) = 34.15, p<0.001; striatum: F(2,21) = 39.16, p<0.001; and hippocampus: F(2,21) = 29.29, p<0.001] (Table 1).

**Table 1:** Fatty acid amide hydrolase (FAAH) activity.

	Frontal cortex	Striatum	Hippocampus
**Vehicle**	600.50 ± 29.49	308.06 ± 15.03	577.88 ± 52.38
**ST4070 (10 mg/kg)**	251.19 ± 61.84**	124.06 ± 30.27**	258.69 ± 34.54**
**ST4070 (30 mg/kg)**	145.88 ± 16.75**	65.50 ± 9.23**	169.00 ± 28.18**

Data are expressed as mean ± SEM (pmol/min per mg protein). Animals were orally administered ST4070 (or the corresponding vehicle) 60 min before the behavioural test (LD box, see text for details). Immediately after the behavioural tests, animals were sacrificed and brain regions rapidly dissected on ice (n = 8 per experimental group). One-way ANOVA followed by Tukey *post-hoc* comparisons,

** p < 0.001.

**Fig 3:**
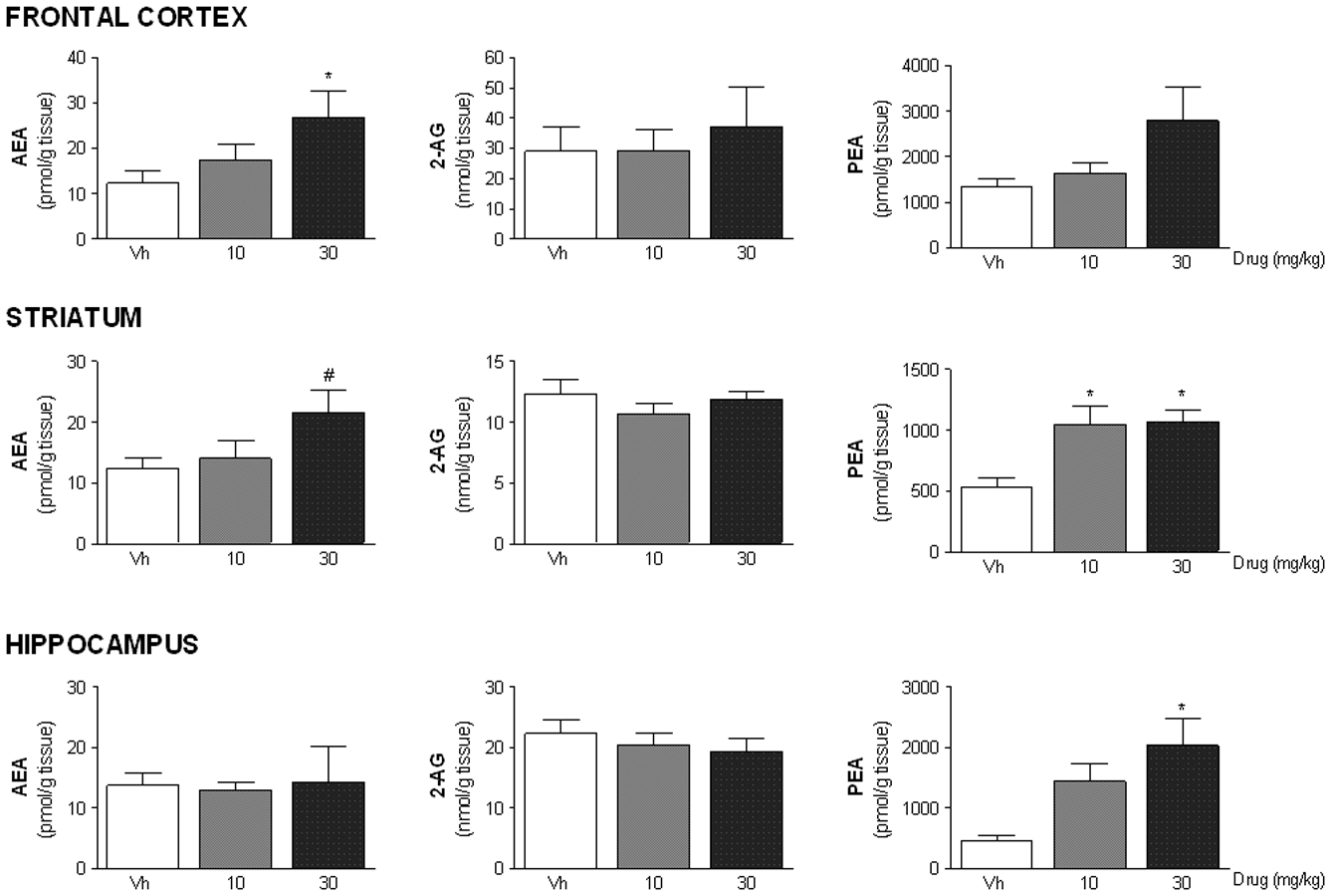
ST4070 effects on brain endocannabinoid content. Data are expressed as mean ± SEM. Rats were orally administered ST4070 (10 or 30 mg/kg, 60 min before testing), and were challenged in the LD box for 10 min. Immediately after testing, animals were sacrificed and brain regions rapidly dissected. Levels of anandamide (AEA), 2-arachidonoylglycerol (2-AG) and *N*-palmitoylethanolamine (PEA) were measured in specific brain regions (frontal cortex, striatum and hippocampus) (n = 8 per experimental group). One-way ANOVA a followed by Tukey *post-hoc* comparisons, * p = 0.05 vs. vehicle; # p = 0.06 *vs*. vehicle.

The inhibitory activity of ST4070 on FAAH was further supported by the evaluation of brain AEA levels (Fig 3). The highest dose of ST4070 (30 mg/kg) significantly increased AEA levels in the striatum [F(2,17) = 3.70, p<0.05] and the frontal cortex [F(2,18) = 3.18, p = 0.065]. However, AEA levels within the hippocampus were not modified by oral administration of ST4070 at any dose [F(2,17) = 0.05, ns]. Although FAAH has also been reported to deactivate other eCBs, including 2-AG [27], no changes in 2-AG content were observed in the frontal cortex [F(2,20) = 0.23, ns], striatum [F(2,19) = 0.84, ns] or hippocampus [F(2,20) = 0.50, ns]. FAAH can also increase the levels of PEA [28]. Consistently, ST4070 significantly increased PEA levels within the striatum [F(2,18) = 6.33, p < 0.01] and the hippocampus [F(2,19) = 5.27, p < 0.05]. *Post-hoc* comparisons indicated that ST4070 at the two doses administered significantly elevated PEA levels within the striatum (p<0.05), whereas in the hippocampus statistical significance was reached only at the highest dose (p<0.05). In addition, a similar trend, yet not significant, could be observed for PEA content within the frontal cortex [F(2,21) = 2.60, ns] (Data in S3 Table).

## Discussion

A link between the anxiety-related effects of FAAH inhibitors and their ability to enhance endogenous AEA signalling has been already proposed [12,29]. Here, we support this concept by using a novel FAAH inhibitor that elicits anxiolytic-like responses when administered orally.

### ST4070 as a potential anxiolytic agent in animal models

Present results indicate that ST4070 modulates anxiety-like responses in both the EPM and the LD box, animal models based on rodents innate general avoidance behaviours [30,31]. Present results are in accordance with previous studies in which other FAAH inhibitors induced anxiolytic-like responses in rodents. In particular, when tested in the elevated plus-maze [32,33,34,35], zero maze [12], LD box [11,36,37], as well as in the marble burying assay [38]. Our data indicate that the highest dose (30 mg/kg) of the novel compound ST4070, orally administered to mice and rats, induced anxiolytic-like responses in the elevated plus-maze and in the LD box; thus, ST4070 arises as a novel eCB-based anxiolytic drug orally effective in rodents. More recently URB597 has been reported to shape behavioural responses to challenges [39], thus it would also be interesting to evaluate the influence of our novel compound, ST4070, on behavioural responses induced by challenges of different intensity.

### ST4070 as a modulator of the eCB system

Here, we have reported that ST4070, through the inhibition of FAAH activity, elevates AEA levels in brain regions critically involved in the control of anxiety and stress response, such as frontal cortex and dorsal striatum [17]. Interestingly, it is now apparent that anxiety does not depend on specific brain areas performing unique functions, but it is rather considered an emerging property of interacting brain regions [40]. Present findings highlight the relevance of the eCB system within these two brain areas (frontal cortex and striatum) in which the control of anxiety-related responses may probably relay on behavioural inhibition under conflict situations [40].

In a previous report ST4070 was identified as a potent reversible inhibitor of FAAH [15]. Much alike other peripherally administered FAAH inhibitors [12,13,41], oral ST4070 effectively reached its pharmacological target in the central nervous system, where it was able to produce a consistent inhibition of FAAH activity within anxiety-relevant brain regions. Despite basal FAAH activity levels differ between brain regions, ST4070 was able to inhibit FAAH activity in all the brain regions analyzed. Remarkably, the inhibitory potency of ST4070 was similar in all brain regions analyzed, being ~50% upon administration of the lowest dose (10 mg/kg), and ~75% upon administration of the highest dose (30 mg/kg). Consistently with FAAH inhibition, an increase in AEA content was observed in the striatum and the frontal cortex, although no changes in AEA levels were observed within the hippocampus. The lack of correlation between FAAH activity and AEA levels in the hippocampus seems noteworthy, and extends previous data on chronic administration of URB597 [13]. A reduction in AEA mobilization could represent a possible underlying mechanism [13]. In addition, alternative catabolic pathways for AEA [42,43] may also compensate for reduced FAAH activity in the hippocampus. ST4070 did not modify 2-AG levels, probably because enzymes other than FAAH are known to be the main responsible for 2-AG degradation. Indeed, MAGL [44] and alfa-beta-hydrolase domain 6 (ABHD6) [45] clearly play a prominent role in 2-AG hydrolysis within the brain.

Furthermore, our data suggest that AEA levels in the hippocampus are not involved in the anxiolytic properties of ST4070, although a contribution of FAAH inhibition in this brain region cannot be ruled out, based on recent data with URB597 [46]. Indeed the hippocampal eCB system, mainly through a role in neural plasticity, has been recognized as pivotal in emotional responses [47]. Overall, we propose that the profile of FAAH activity and AEA levels in different anxiety-relevant brain regions may change time-dependently, in order to provide an appropriate response to a certain type of conflict. For instance, an early integration of multimodal sensory information, mediated within the hippocampus, is needed prior to the behavioural inhibition directed by the striatum and the prefrontal cortex [17]. The timing of activation of the diverse neuroanatomical substrates mediating the anxiolytic-like effects of ST4070 remains to be elucidated. Incidentally, stress exposure seems to be crucial for the impact of eCB signalling on anxiety-related responses [48]. Therefore, future studies on the anxiolytic-like properties of eCB-targeted drugs should include both unstressed and stressed subjects.

Last but not least, it should also be noted that, unlike most of FAAH inhibitors with potential as therapeutics, ST4070 reversibly inhibits enzyme activity [15]. To date, only a few reversible FAAH inhibitors have been described in the literature, i.e. the α-ketoheterocycle OL-135 [49], and the covalent but slowly reversible piperazine urea JNJ-1661010 developed by Johnson & Johnson [50]. Although further research is still needed, reversible inhibitors might offer advantages over irreversible blockers as lead compounds for drug design [51], and possibly also for the management of anxiety-related disorders.

## Conclusions

The new enol-carbamate ST4070 that affords potent and reversible inhibition of FAAH *in vivo*, enhances the endogenous eCB tone in specific brain regions engaged in emotional control, and induces remarkable anxiolytic-like behaviours in rodents. Although research on the neural substrates and pharmacokinetics of the anxiolytic-like effects of ST4070 in rodents is still needed, the present investigation opens new avenues to the development and further evaluation of a new family of FAAH inhibitors as drugs to be tested in clinical trials for the management of anxiety and mood disorders in humans.

## Supporting Information

S1 Table Elevated Plus Maze (EPM) in mice. (Individual data).
(XLS)

S2 Table Light-Dark (LD) Box in rats. (Individual data).
(XLS)

S3 Table FAAH activity and brain endocannabinoid (eCB) content. (Individual data).
(XLS)

## References

[1] Millan MJ, Goodwin GM, Meyer-Lindenberg A, Ove Ogren S. Learning from the past and looking to the future: Emerging perspectives for improving the treatment of psychiatric disorders. Eur Neuropsychopharmacol. 2015 Epub 2015/04/04. S0924-977X(15)00017-6 [pii] 10.1016/j.euroneuro.2015.01.016 . 25836356

[2] Cryan JF, Sweeney FF. The age of anxiety: role of animal models of anxiolytic action in drug discovery. Br J Pharmacol. 2011;164(4):1129–61. Epub 2011/05/07. 10.1111/j.1476-5381.2011.01362.x 21545412 PMC3229755

[3] Crippa JA, Zuardi AW, Martin-Santos R, Bhattacharyya S, Atakan Z, McGuire P, et al Cannabis and anxiety: a critical review of the evidence. Hum Psychopharmacol. 2009;24(7):515–23. Epub 2009/08/21. 10.1002/hup.1048 . 19693792

[4] Viveros MP, Marco EM, File SE. Endocannabinoid system and stress and anxiety responses. Pharmacol Biochem Behav. 2005;81(2):331–42. Epub 2005/06/02. S0091-3057(05)00134-6 [pii] 10.1016/j.pbb.2005.01.029 . 15927244

[5] Marco EM, Garcia-Gutierrez MS, Bermudez-Silva FJ, Moreira FA, Guimaraes F, Manzanares J, et al Endocannabinoid system and psychiatry: in search of a neurobiological basis for detrimental and potential therapeutic effects. Front Behav Neurosci. 2011;5:63 Epub 2011/10/19. 10.3389/fnbeh.2011.00063 22007164 PMC3186912

[6] Piomelli D. The molecular logic of endocannabinoid signalling. Nat Rev Neurosci. 2003;4(11):873–84. Epub 2003/11/05. 10.1038/nrn1247 nrn1247 [pii]. . 14595399

[7] Maccarrone M, Guzman M, Mackie K, Doherty P, Harkany T. Programming of neural cells by (endo)cannabinoids: from physiological rules to emerging therapies. Nat Rev Neurosci. 2014;15(12):786–801. Epub 2014/11/21. nrn3846 [pii] 10.1038/nrn3846 . 25409697 PMC4765324

[8] Pertwee RG. Emerging strategies for exploiting cannabinoid receptor agonists as medicines. Br J Pharmacol. 2009;156(3):397–411. Epub 2009/02/20. BPH048 [pii] 10.1111/j.1476-5381.2008.00048.x 19226257 PMC2697681

[9] Batista LA, Gobira PH, Viana TG, Aguiar DC, Moreira FA. Inhibition of endocannabinoid neuronal uptake and hydrolysis as strategies for developing anxiolytic drugs. Behav Pharmacol. 2014;25(5–6):425–33. Epub 2014/08/02. 10.1097/FBP.0000000000000073 00008877-201409000-00009 [pii]. . 25083569

[10] Ahn K, Johnson DS, Mileni M, Beidler D, Long JZ, McKinney MK, et al Discovery and characterization of a highly selective FAAH inhibitor that reduces inflammatory pain. Chem Biol. 2009;16(4):411–20. Epub 2009/04/25. S1074-5521(09)00080-5 [pii] 10.1016/j.chembiol.2009.02.013 19389627 PMC2692831

[11] Rutkowska M, Jamontt J, Gliniak H. Effects of cannabinoids on the anxiety-like response in mice. Pharmacol Rep. 2006;58(2):200–6. Epub 2006/05/17. . 16702621

[12] Kathuria S, Gaetani S, Fegley D, Valino F, Duranti A, Tontini A, et al Modulation of anxiety through blockade of anandamide hydrolysis. Nat Med. 2003;9(1):76–81. Epub 2002/12/04. 10.1038/nm803 nm803 [pii]. . 12461523

[13] Bortolato M, Mangieri RA, Fu J, Kim JH, Arguello O, Duranti A, et al Antidepressant-like activity of the fatty acid amide hydrolase inhibitor URB597 in a rat model of chronic mild stress. Biol Psychiatry. 2007;62(10):1103–10. Epub 2007/05/22. S0006-3223(06)01552-6 [pii] 10.1016/j.biopsych.2006.12.001 . 17511970

[14] Kwilasz AJ, Abdullah RA, Poklis JL, Lichtman AH, Negus SS. Effects of the fatty acid amide hydrolase inhibitor URB597 on pain-stimulated and pain-depressed behavior in rats. Behav Pharmacol. 2014;25(2):119–29. Epub 2014/03/04. 10.1097/FBP.0000000000000023 24583930 PMC3963812

[15] Gattinoni S, De Simone C, Dallavalle S, Fezza F, Nannei R, Amadio D, et al Enol carbamates as inhibitors of fatty acid amide hydrolase (FAAH) endowed with high selectivity for FAAH over the other targets of the endocannabinoid system. ChemMedChem. 2010;5(3):357–60. Epub 2010/01/30. 10.1002/cmdc.200900472 . 20112328

[16] Gattinoni S, Simone CD, Dallavalle S, Fezza F, Nannei R, Battista N, et al A new group of oxime carbamates as reversible inhibitors of fatty acid amide hydrolase. Bioorg Med Chem Lett. 2010;20(15):4406–11. Epub 2010/07/02. S0960-894X(10)00824-3 [pii] 10.1016/j.bmcl.2010.06.050 . 20591666

[17] Canteras NS, Resstel LB, Bertoglio LJ, Carobrez Ade P, Guimaraes FS. Neuroanatomy of anxiety. Curr Top Behav Neurosci. 2010;2:77–96. Epub 2011/02/11. . 21309107 10.1007/7854_2009_7

[18] Rodgers RJ, Cao BJ, Dalvi A, Holmes A. Animal models of anxiety: an ethological perspective. Braz J Med Biol Res. 1997;30(3):289–304. Epub 1997/03/01. . 9246227 10.1590/s0100-879x1997000300002

[19] Bourin M, Hascoet M. The mouse light/dark box test. Eur J Pharmacol. 2003;463(1–3):55–65. Epub 2003/02/26. S0014299903012743 [pii]. . 12600702 10.1016/s0014-2999(03)01274-3

[20] Campos AC, Fogaca MV, Aguiar DC, Guimaraes FS. Animal models of anxiety disorders and stress. Rev Bras Psiquiatr. 2013;35 Suppl 2:S101–11. Epub 2013/12/07. S1516-44462013000600006 [pii] 10.1590/1516-4446-2013-1139 . 24271222

[21] Steimer T. Animal models of anxiety disorders in rats and mice: some conceptual issues. Dialogues Clin Neurosci. 2011;13(4):495–506. Epub 2012/01/26. 22275854 10.31887/DCNS.2011.13.4/tsteimer PMC3263396

[22] Carobrez AP, Bertoglio LJ. Ethological and temporal analyses of anxiety-like behavior: the elevated plus-maze model 20 years on. Neurosci Biobehav Rev. 2005;29(8):1193–205. Epub 2005/08/09. S0149-7634(05)00068-0 [pii] 10.1016/j.neubiorev.2005.04.017 . 16084592

[23] Steimer T, Driscoll P. Divergent stress responses and coping styles in psychogenetically selected Roman high-(RHA) and low-(RLA) avoidance rats: behavioural, neuroendocrine and developmental aspects. Stress. 2003;6(2):87–100. Epub 2003/05/31. 10.1080/1025389031000111320 UE4X34PWXAQ94T1T [pii]. . 12775328

[24] Francavilla F, Battista N, Barbonetti A, Vassallo MR, Rapino C, Antonangelo C, et al Characterization of the endocannabinoid system in human spermatozoa and involvement of transient receptor potential vanilloid 1 receptor in their fertilizing ability. Endocrinology. 2009;150(10):4692–700. Epub 2009/07/18. en.2009-0057 [pii] 10.1210/en.2009-0057 . 19608651

[25] Kilfoil T, Michel A, Montgomery D, Whiting RL. Effects of anxiolytic and anxiogenic drugs on exploratory activity in a simple model of anxiety in mice. Neuropharmacology. 1989;28(9):901–5. Epub 1989/09/01. . 2572995 10.1016/0028-3908(89)90188-3

[26] Young R, Johnson DN. A fully automated light/dark apparatus useful for comparing anxiolytic agents. Pharmacol Biochem Behav. 1991;40(4):739–43. Epub 1991/12/01. . 1687762 10.1016/0091-3057(91)90078-g

[27] Ahn K, Johnson DS, Cravatt BF. Fatty acid amide hydrolase as a potential therapeutic target for the treatment of pain and CNS disorders. Expert Opin Drug Discov. 2009;4(7):763–84. Epub 2010/06/15. 10.1517/17460440903018857 20544003 PMC2882713

[28] Hansen HS. Palmitoylethanolamide and other anandamide congeners. Proposed role in the diseased brain. Exp Neurol. 2010;224(1):48–55. Epub 2010/04/01. S0014-4886(10)00107-X [pii] 10.1016/j.expneurol.2010.03.022 . 20353771

[29] Piomelli D, Tarzia G, Duranti A, Tontini A, Mor M, Compton TR, et al Pharmacological profile of the selective FAAH inhibitor KDS-4103 (URB597). CNS Drug Rev. 2006;12(1):21–38. Epub 2006/07/13. CNS21 [pii] 10.1111/j.1527-3458.2006.00021.x . 16834756 PMC6741741

[30] Crawley J, Goodwin FK. Preliminary report of a simple animal behavior model for the anxiolytic effects of benzodiazepines. Pharmacol Biochem Behav. 1980;13(2):167–70. Epub 1980/08/01. . 6106204 10.1016/0091-3057(80)90067-2

[31] Hogg S. A review of the validity and variability of the elevated plus-maze as an animal model of anxiety. Pharmacol Biochem Behav. 1996;54(1):21–30. Epub 1996/05/01. 0091-3057(95)02126-4 [pii]. . 8728535 10.1016/0091-3057(95)02126-4

[32] Moreira FA, Lutz B. The endocannabinoid system: emotion, learning and addiction. Addict Biol. 2008;13(2):196–212. Epub 2008/04/22. ADB104 [pii] 10.1111/j.1369-1600.2008.00104.x . 18422832

[33] Naidu PS, Varvel SA, Ahn K, Cravatt BF, Martin BR, Lichtman AH. Evaluation of fatty acid amide hydrolase inhibition in murine models of emotionality. Psychopharmacology (Berl). 2007;192(1):61–70. Epub 2007/02/07. 10.1007/s00213-006-0689-4 . 17279376

[34] Patel S, Hillard CJ. Pharmacological evaluation of cannabinoid receptor ligands in a mouse model of anxiety: further evidence for an anxiolytic role for endogenous cannabinoid signaling. J Pharmacol Exp Ther. 2006;318(1):304–11. Epub 2006/03/30. jpet.106.101287 [pii] 10.1124/jpet.106.101287 . 16569753

[35] Micale V, Cristino L, Tamburella A, Petrosino S, Leggio GM, Drago F, et al Anxiolytic effects in mice of a dual blocker of fatty acid amide hydrolase and transient receptor potential vanilloid type-1 channels. Neuropsychopharmacology. 2009;34(3):593–606. Epub 2008/06/27. npp200898 [pii] 10.1038/npp.2008.98 . 18580871

[36] Moreira FA, Kaiser N, Monory K, Lutz B. Reduced anxiety-like behaviour induced by genetic and pharmacological inhibition of the endocannabinoid-degrading enzyme fatty acid amide hydrolase (FAAH) is mediated by CB1 receptors. Neuropharmacology. 2008;54(1):141–50. Epub 2007/08/22. S0028-3908(07)00214-6 [pii] 10.1016/j.neuropharm.2007.07.005 . 17709120

[37] Scherma M, Medalie J, Fratta W, Vadivel SK, Makriyannis A, Piomelli D, et al The endogenous cannabinoid anandamide has effects on motivation and anxiety that are revealed by fatty acid amide hydrolase (FAAH) inhibition. Neuropharmacology. 2008;54(1):129–40. Epub 2007/10/02. S0028-3908(07)00268-7 [pii] 10.1016/j.neuropharm.2007.08.011 17904589 PMC2213536

[38] Kinsey SG, O'Neal ST, Long JZ, Cravatt BF, Lichtman AH. Inhibition of endocannabinoid catabolic enzymes elicits anxiolytic-like effects in the marble burying assay. Pharmacol Biochem Behav. 2011;98(1):21–7. Epub 2010/12/15. S0091-3057(10)00365-5 [pii] 10.1016/j.pbb.2010.12.002 21145341 PMC3034086

[39] Haller J, Aliczki M, Pelczer KG, Spitzer K, Balogh Z, Kantor S. Effects of the fatty acid amide hydrolase inhibitor URB597 on coping behavior under challenging conditions in mice. Psychopharmacology (Berl). 2014;231(3):593–601. Epub 2013/09/17. 10.1007/s00213-013-3273-8 . 24037493

[40] Morgane PJ, Galler JR, Mokler DJ. A review of systems and networks of the limbic forebrain/limbic midbrain. Prog Neurobiol. 2005;75(2):143–60. Epub 2005/03/24. S0301-0082(05)00002-X [pii] 10.1016/j.pneurobio.2005.01.001 . 15784304

[41] Moise AM, Eisenstein SA, Astarita G, Piomelli D, Hohmann AG. An endocannabinoid signaling system modulates anxiety-like behavior in male Syrian hamsters. Psychopharmacology (Berl). 2008;200(3):333–46. Epub 2008/06/12. 10.1007/s00213-008-1209-5 18545985 PMC2694060

[42] Okamoto Y, Tsuboi K, Ueda N. Enzymatic formation of anandamide. Vitam Horm. 2009;81:1–24. Epub 2009/08/04. S0083-6729(09)81001-7 [pii] 10.1016/S0083-6729(09)81001-7 . 19647106

[43] Simon GM, Cravatt BF. Characterization of mice lacking candidate N-acyl ethanolamine biosynthetic enzymes provides evidence for multiple pathways that contribute to endocannabinoid production in vivo. Mol Biosyst. 2010;6(8):1411–8. Epub 2010/04/16. 10.1039/c000237b 20393650 PMC2946841

[44] Dinh TP, Carpenter D, Leslie FM, Freund TF, Katona I, Sensi SL, et al Brain monoglyceride lipase participating in endocannabinoid inactivation. Proc Natl Acad Sci U S A. 2002;99(16):10819–24. Epub 2002/07/24. 10.1073/pnas.152334899 152334899 [pii]. 12136125 PMC125056

[45] Marrs WR, Blankman JL, Horne EA, Thomazeau A, Lin YH, Coy J, et al The serine hydrolase ABHD6 controls the accumulation and efficacy of 2-AG at cannabinoid receptors. Nat Neurosci. 2010;13(8):951–7. Epub 2010/07/27. nn.2601 [pii] 10.1038/nn.2601 20657592 PMC2970523

[46] Rivera P, Bindila L, Pastor A, Perez-Martin M, Pavon FJ, Serrano A, et al Pharmacological blockade of the fatty acid amide hydrolase (FAAH) alters neural proliferation, apoptosis and gliosis in the rat hippocampus, hypothalamus and striatum in a negative energy context. Front Cell Neurosci. 2015;9:98 Epub 2015/04/15. 10.3389/fncel.2015.00098 25870539 PMC4375993

[47] Viveros MP, Marco EM, Llorente R, Lopez-Gallardo M. Endocannabinoid system and synaptic plasticity: implications for emotional responses. Neural Plast. 2007;2007:52908 Epub 2007/07/21. 10.1155/2007/52908 17641734 PMC1906867

[48] Kinden R, Zhang X. Cannabinoids & Stress: Impact of HU-210 on behavioral tests of anxiety in acutely stressed mice. Behav Brain Res. 2015;284:225–30. Epub 2015/02/25. S0166-4328(15)00104-7 [pii] 10.1016/j.bbr.2015.02.025 . 25707713

[49] Lichtman AH, Leung D, Shelton CC, Saghatelian A, Hardouin C, Boger DL, et al Reversible inhibitors of fatty acid amide hydrolase that promote analgesia: evidence for an unprecedented combination of potency and selectivity. J Pharmacol Exp Ther. 2004;311(2):441–8. Epub 2004/07/02. 10.1124/jpet.104.069401 jpet.104.069401 [pii]. . 15229230

[50] Keith JM, Apodaca R, Xiao W, Seierstad M, Pattabiraman K, Wu J, et al Thiadiazolopiperazinyl ureas as inhibitors of fatty acid amide hydrolase. Bioorg Med Chem Lett. 2008;18(17):4838–43. Epub 2008/08/12. S0960-894X(08)00868-8 [pii] 10.1016/j.bmcl.2008.07.081 . 18693015

[51] Leung D, Hardouin C, Boger DL, Cravatt BF. Discovering potent and selective reversible inhibitors of enzymes in complex proteomes. Nat Biotechnol. 2003;21(6):687–91. Epub 2003/05/13. 10.1038/nbt826 nbt826 [pii]. . 12740587

